# Meta‐analysis of peritoneal lavage in appendicectomy

**DOI:** 10.1002/bjs5.50118

**Published:** 2018-11-29

**Authors:** E. Gammeri, T. Petrinic, G. Bond‐Smith, A. Gordon‐Weeks

**Affiliations:** ^1^ Department of General Surgery John Radcliffe Hospital Oxford UK; ^2^ Cairns Library, John Radcliffe Hospital Oxford UK; ^3^ Nuffield Department of Surgical Sciences University of Oxford Oxford UK

## Abstract

**Background:**

The use of peritoneal lavage to prevent postoperative intra‐abdominal abscess (IAA) after appendicectomy has been debated widely.

**Methods:**

A systematic review and meta‐analysis of suction alone *versus* lavage for appendicitis was performed to determine the relative benefit of lavage. Primary outcomes were postoperative IAA and wound infection (WI). Inclusion criteria were human studies reporting a comparison of appendicectomy with or without peritoneal lavage.

**Results:**

Eight studies met the inclusion criteria, the majority of which were retrospective. Only three were RCTs. Four studies included analysis only of the paediatric population. The rate of IAA was 1·0–19·5 per cent in patients receiving suction alone and 1·5–18·6 per cent in those having lavage. WI rates were 1·0–29·2 per cent for suction alone and 0·8–20·5 per cent for lavage. The pooled risk difference for IAA was 0·01 (95 per cent c.i. −0·03 to 0·06; *P* = 0·50) and that for WI was 0·00 (−0·05 to 0·05; *P* = 0·98). Analyses of both outcomes indicated a medium degree of heterogeneity between effect estimates with *I* ^2^ values of 71 per cent (*P* = 0·001) and 70 per cent (*P* = 0·010) for IAA and WI respectively.

**Conclusion:**

There is no evidence of benefit of lavage over suction for postoperative infective complications, and no individual study demonstrated a significant benefit in patients receiving lavage.

## Introduction

Acute appendicitis is one of the commonest causes of emergency surgical admission. Throughout the Western world, laparoscopy has become the favoured approach for appendicectomy^1^ ^2^. Proposed benefits over open appendicectomy include reduced incidence of wound infection (WI), better visualization, reduced postoperative pain, shorter hospital stay, earlier return to work and reduced incidence of incisional hernia^3^. However, intra‐abdominal abscess (IAA) remains one of the most problematic complications following appendicectomy; thus, techniques that reduce IAA incidence will impact significantly upon surgical outcomes.

The use of peritoneal lavage with or without antibiotic solution to prevent IAA has been debated widely^4^, ^5^, ^6^, ^7^. Several studies^8^ ^9^ have failed to identify a significant benefit for peritoneal lavage in patients with peritonitis from various sources. Nonetheless, proponents of lavage argue that thoroughly irrigating the peritoneum dilutes the bacterial load and thereby the risk of postoperative septic complications^4^. The counter argument is that lavage disperses an otherwise localized bacterial contaminant throughout the peritoneal cavity, predisposing to interloop abscess. Potential complications of peritoneal lavage, particularly through the use of antibiotic solutions, include the formation of intra‐abdominal adhesions^10^ and serositis leading to ascites formation^11^.

Laparoscopic lavage has been used as a primary treatment for other causes of intra‐abdominal sepsis, with most notable success in localized diverticular perforation^12^, ^13^, ^14^. Although this approach has gained popularity within the emergency surgical community, it carries a reasonably high risk of postoperative IAA and a higher risk of reoperation than resectional surgery^15^ ^16^, indicating that lavage may not be effective in preventing IAA when the bacterial load is significant.

Several RCTs^17^, ^18^, ^19^ have compared appendicectomy performed with suction alone with various methods of lavage; however, no systematic analysis of such studies has been performed. The aim of this systematic review was to determine the benefit of lavage for appendicitis *versus* suction alone, focusing particularly on postoperative IAA and WI.

## Methods

Meta‐analysis was performed in accordance with PRISMA guidance^20^. Owing to the inclusion of retrospective cohort studies in this analysis, the guidance from the Meta‐analysis Of Observational Studies in Epidemiology (MOOSE) group^21^ was also followed.

### Identification of studies for inclusion

MEDLINE (PubMed) and Embase (Ovid) were searched on 11 September 2017 by a healthcare librarian. Keywords included appendicectomy, appendectomy, appendicitis, lavage, suction, irrigation, wash, washout and aspiration. Keywords were combined using Boolean operators and MeSH (Medical Subject Headings) terms were exploded throughout.

All abstracts generated from the search were read, and full‐text publications of abstracts meeting the search criteria on initial screening were reviewed to confirm whether the inclusion and exclusion criteria had been met. References from studies meeting these criteria at the full‐text stage were hand‐searched to identify further studies for inclusion.

### Inclusion and exclusion criteria

Studies in humans reporting a comparison between groups of patients undergoing appendicectomy with or without peritoneal lavage were included. RCTs and observational studies were included, as were studies of paediatric populations. Both open and laparoscopic approaches were included. Studies were included only if some, or all, of the patients were treated for complex appendicitis (localized or diffuse peritonitis). Animal studies were excluded, as were case reports and studies in non‐English‐language journals.

### Data extraction and statistical analysis

For each included study, data were extracted by two authors and discrepancies resolved by discussion and further joint review.

Outcome results from dichotomous variables were combined using the Mantel–Haenszel method and continuous variables using inverse variance; pooled estimates are presented as risk difference (RD) and mean difference (MD) respectively. All effect estimates were calculated using the random‐effects model (DerSimonian and Laird) as significant heterogeneity between individual study results was expected given their variability in design and demographics.

Statistical analysis was performed using Review Manager version 5.3 (The Cochrane Collaboration, Oxford, UK). Heterogeneity between study effect estimates was assessed using the Cochran *Q* statistic (χ^2^ test) and *I* ^2^. Heterogeneity was considered high, medium or low if 75 per cent or above, 50–74 per cent, or less than 50 per cent respectively^22^. Funnel plots were produced for IAA and WI outcomes and assessed visually. *P* < 0·050 was considered statistically significant.

### Assessment of publication quality

Two authors independently assessed the quality of included studies using the Quality Assessment of Controlled Interventional Studies and Quality Assessment Tool for Observational Cohort and Cross‐Sectional Studies tools for RCTs or observational studies respectively (https://www.nhlbi.nih.gov/health‐pro/guidelines/in‐develop/cardiovascular‐risk‐reduction/tools). Discrepancies were resolved by discussion and joint analysis.

## Results

**Figure 1:**
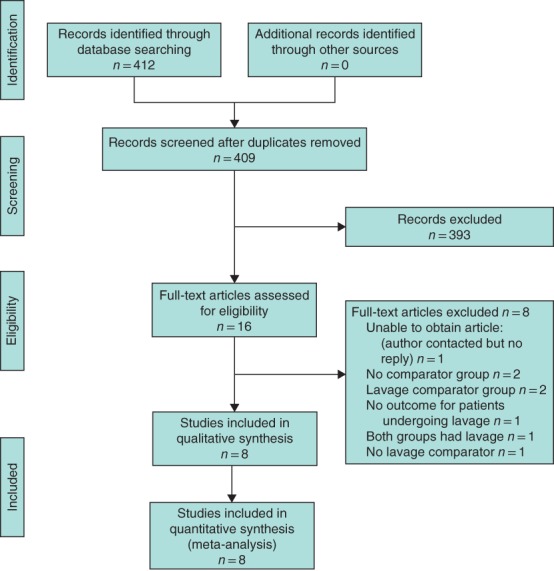
PRISMA diagram for the review

A total of eight studies including 3034 patients met the inclusion criteria (*Fig*. 1). All studies reported postoperative IAA, five studies (821 patients) reported WI rate, five (803 patients) reported length of hospital stay, and three studies (561 patients) reported duration of surgery.

One full‐text publication^4^ that met the inclusion criteria at the abstract stage could not be located electronically or after contact with both the publisher and author, and so was not included in the data analysis.

### Study demographics and design

Study demographics are summarized in *Table* *S1* (supporting information). The study interval ranged from 1965 to 2016, and thus encompasses the prelaparoscopic era to the present day, including studies from healthcare centres across four continents. Three of the included studies^17^, ^18^, ^19^ were RCTs; the remainder were retrospective cohort studies. Four studies^19^ ^23^, ^24^, ^25^ included analysis only of the paediatric population, two^24^ ^25^ of which were performed entirely using open surgery. The age of the population was poorly reported throughout. There was a high degree of variation between pathological findings at surgery, with some studies^18^ ^19^, ^23^, ^24^, ^25^ analysing the outcomes only of patients with complex appendicitis (perforation, gangrene, localized abscess)_,_ whereas the remaining studies included patients with simple appendicitis (inflammation in the appendix alone). In some studies^17^ ^18^ drains were never used as part of local institutional policy, whereas drains were used in the remaining studies at the discretion of the operating surgeon.

A summary of the relevant design characteristics of the included studies is shown in *Table S2* (supporting information). The intraoperative technique varied little between studies of laparoscopic surgery, with a three‐port technique used in all cases; no study employed single‐port or natural‐orifice surgery. Notably, the methods of lavage varied between studies. In three studies^18^ ^24^, ^25^ copious lavage throughout all four quadrants was employed, whereas in others^17^ ^19^, ^23^ ^26^ the decision between four‐quadrant and local lavage was made at the discretion of the surgeon. In no study was the number of patients undergoing four‐quadrant or local lavage reported. Three studies^7^ ^17^, ^19^ used preoperative antibiotics routinely and four^19^ ^23^, ^24^ ^26^ used postoperative antibiotics. Antibiotic usage was not reported in two studies^18^ ^25^. Only two of the studies^17^ ^23^ included details on how to define the primary outcome measure IAA and only one study^23^ included details on how to define WI (*Table S2*, supporting information).

### Outcome analysis

The rate of IAA was 1·5–18·5 per cent overall, 1·0–19·5 per cent in patients receiving suction alone, and 1·5–18·6 per cent in those undergoing lavage. WI rates ranged from 1·5 to 23·8 per cent overall, 1·0 to 29·2 per cent in patients receiving suction alone, and 0·8 to 20·5 per cent in those having lavage.

**Figure 2:**
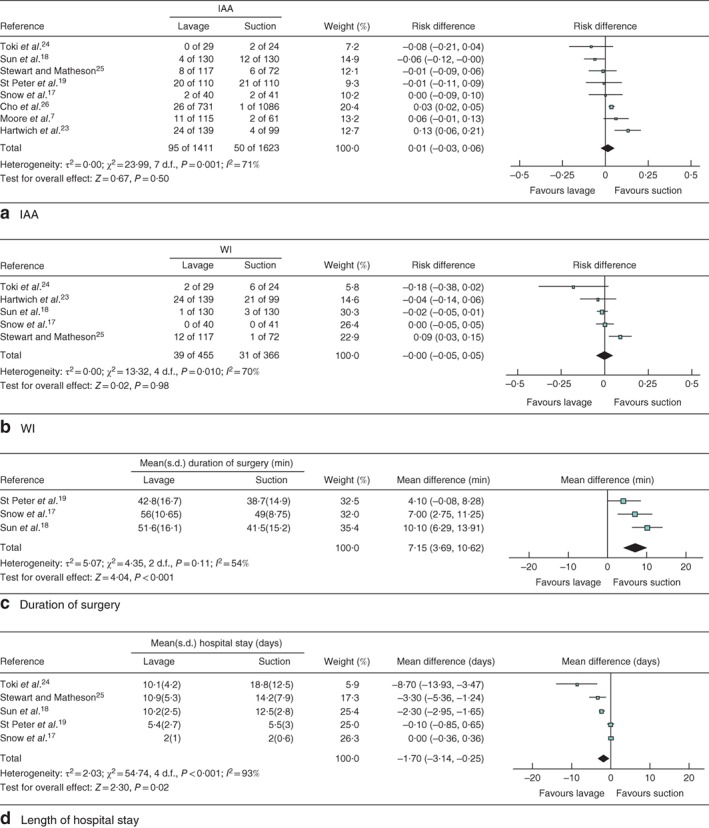
Forest plots of postoperative intra‐abdominal abscess, wound infection, duration of surgery and length of hospital stay. **a** Intra‐abdominal abscess (IAA), **b** wound infection (WI), **c** duration of surgery, **d** length of hospital stay. **a,b** Mantel–Haenszel and **c,d** inverse variance random‐effects models were used for meta‐analysis. **a,b** Risk differences and **c,d** mean differences are shown with 95 per cent confidence intervals.

The pooled RD for IAA was 0·01 (95 per cent c.i. −0·03 to 0·06; *P* = 0·50) (*Fig*. 2 *a*), and that for WI was 0·00 (−0·05 to 0·05; *P* = 0·98) (*Fig*. 2 *b*). Analyses of both outcomes indicated a medium degree of heterogeneity between effect estimates, with *I* ^2^ values of 71 per cent (*P* = 0·001) and 70 per cent (*P* = 0·010) for IAA and WI respectively (*Fig*. 2 *a,b*).

The pooled MD in duration of surgery was 7·15 (95 per cent c.i. 3·69 to 10·62) min (*P* < 0·001) in favour of suction over lavage, whereas the MD in hospital stay was −1·70 (−3·14 to −0·25) days (*P* = 0·02) in favour of lavage over suction (*Fig*. 2 *c,d*). Significant heterogeneity existed in the effect sizes between studies included in these analyses.

### Assessment of study quality

The assessment of study quality demonstrates that included study quality was reasonable, but there were major shortcomings, particularly in the practice of blinding of both the outcome assessor or participant to the intervention arm (*Table* 1).

**Table 1:** Quality assessment of controlled intervention, observational cohort and cross‐sectional studies

	Sun *et al*.^18^	Snow *et al*.^17^	Cho *et al*.^26^	Hartwich *et al*.^23^	St Peter *et al*.^19^	Moore *et al*.^7^	Toki *et al*.^24^	Stewart and Matheson^25^
1. Was the research question or objective in this paper clearly stated?	Yes	Yes	Yes	Yes	Yes	?	Yes	Yes
2. Was the study population clearly specified and defined?	Yes	Yes	Yes	Yes	Yes	?	Yes	Yes
3. Was the participation rate of eligible persons at least 50%?	Yes	Yes	Yes	Yes	Yes	?	Yes	Yes
4. Were all the subjects selected or recruited from the same or similar populations? Were inclusion and exclusion criteria for being in the study prespecified and applied uniformly to all participants?	Yes	Yes	Yes	Yes	Yes	?	Yes	Yes
5. Study described as randomized?	Yes	Yes	No	No	Yes	No	No	No
6. Randomization method adequate?	Yes	Yes	n.a.	n.a.	Yes	?	n.a.	n.a.
7. Was the treatment allocation concealed (so that assignments could not be predicted)?	No	No	n.a.	n.a.	Yes	?	n.a.	n.a.
8. Were study participants and providers blinded to treatment group assignment?	No	No	n.a.	n.a.	Yes	?	n.a.	n.a.
9. Were the people assessing the outcomes blinded to the participants' group assignments?	No	No	n.a.	n.a.	Yes	?	n.a.	n.a.
10. Were the groups similar at baseline on important characteristics that could affect outcomes (e.g. demographics, risk factors, co‐morbid conditions)?	Yes	Yes	n.a.	n.a.	No	?	n.a.	n.a.
11. Was the overall dropout rate from the study at endpoint 20% or lower than the number allocated to treatment?	Yes	Yes	n.a.	n.a.	Yes	?	n.a.	n.a.
12. Was the differential dropout rate (between treatment groups) at endpoint 15% or less?	Yes	Yes	n.a.	n.a.	Yes	?	n.a.	n.a.
13. Was there high adherence to the intervention protocols for each treatment group?	Yes	Yes	n.a.	n.a.	Yes	?	n.a.	n.a.
14. Were other interventions avoided or similar in the groups (e.g. similar background treatments)?	No	No	n.a.	n.a.	Yes	?	n.a.	n.a.
15. Were the outcome measures (dependent variables) clearly defined, valid, reliable and implemented consistently across all study participants?	Yes	Yes	Yes	Yes	Yes	?	Yes	Yes
16. Were the outcome assessors blinded to the exposure status of participants?	No	No	No	No	No	?	No	No
17. Was loss to follow‐up after baseline 20% or less?	Yes	Yes	Yes	Yes	Yes	?	Yes	n.a.

n.a., Not available.

## Discussion

Acute appendicitis can be classified as simple (inflammation confined to the appendix and not involving the peritoneum) or complex (localized or diffuse peritonitis)^27^. As the rationale for lavage after appendicectomy is to dilute bacterial contamination to reduce the rate of IAA, it is illogical to perform lavage in the setting of simple appendicitis where, by definition, bacterial contamination outside the appendix is minimal or absent. With regard to complex appendicitis, despite the potential for the development of florid, four‐quadrant peritonitis, the majority of cases are limited to right iliac fossa peritonitis alone. A key argument against lavage is therefore to prevent spread of infective organisms throughout the peritoneal cavity in the setting of otherwise localized sepsis. As such, the patient population where equipoise exists with regard to lavage is those with complex appendicitis.

The key finding from the pooled analysis presented here is that there is no evidence of benefit for lavage over suction for postoperative infective complications and, importantly, no single study demonstrated a significant benefit for patients who had lavage. Although the superiority trials included are by their nature unable to prove equivalence between lavage and suction, the inclusion of the bioequivalence study by Snow and colleagues^17^, demonstrating that lavage and suction are equivalent to a margin of 15 per cent, supports the notion that lavage provides no additional benefit over suction. This trial was small, and no doubt more expansive trials are required to confirm this; however, the present meta‐analysis provides the best available evidence to date that lavage does not reduce septic complications in patients undergoing appendicectomy.

Interestingly, there was a significant reduction in postoperative hospital stay in patients who received lavage. This finding is intriguing, although further investigation of its causation is outside the scope of this meta‐analysis. However, it may result from postoperative complications such as pain or ileus that were not examined in the included studies but may be more prevalent in patients who have suction alone. This raises the possibility that, although lavage may not prevent overt septic complications, it may provide other benefits that reduce postoperative stay, potentially by diluting bacterial contaminants that, whilst not sufficient to cause IAA, may predispose to ileus. Further investigation in this area is clearly warranted.

The key strengths of this meta‐analysis are its rigorous search strategy enabling inclusion of a relatively large number of patients, most treated for complex appendicitis, thereby addressing the population where equipoise exists. For the majority of patients, the method of lavage was well described and, although it varied between studies with regard to the solutions used, their volumes and application, this does not affect the ability of the study to assess the biological relevance of lavage for reduction of postoperative IAA or WI.

Despite this, the data presented need to be interpreted with some caution. First, certain confounding factors that contribute to postoperative sepsis, including obesity^28^, diabetes mellitus^29^ and immunosuppression, were inadequately reported by the included studies and may have affected the effect estimates. Further, significant heterogeneity was detected in the magnitude of the effect estimates for all outcome measures studied. This is partly attributable to differences in the populations studied, the operative techniques employed, and the methodologies used in each study. For example, some studies analysed paediatric populations alone and others studied exclusively open or laparoscopic approaches.

The inclusion of patients with simple appendicitis by some studies may have contributed to the heterogeneity of effect estimates. As the primary interest is in the benefit of lavage for patients with complex appendicitis, a subgroup analysis could provide informative results; however, given the small number of studies that would meet the inclusion criteria, these results would be heterogeneous and therefore unlikely to generate clinically meaningful data. A further trial in a clearly defined population is thus indicated to assess definitively the benefit of lavage in complex appendicitis.

As evidence of heterogeneity between studies, the overall incidence of postoperative IAA varied significantly. This may be a true variation or could relate to differences in the definition of IAA between studies. Indeed, the definitions used for septic outcome measures were not reported and so it is unclear whether some studies included only clinically relevant postoperative IAA or whether others used routine postoperative imaging, and which clinical parameters in particular led the surgeon to investigate further. This is of relevance given that retained fluid used for lavage could be mistaken for IAA in patients undergoing routine postoperative imaging. Furthermore, there was no attempt to determine whether there was an association between intervention and degree of IAA severity. This information is important, as it is possible that, although the rate of radiologically detected IAA does not differ between lavage and suction, the severity of IAA may be greater in one group. For outcome measures that involve postoperative complications, efforts should be made at stratification based on recognized criteria so that comparison across studies is possible.

## Disclosure

The authors declare no conflict of interest.

## Supporting information


**Table S1** Study demographics
**Table S2** Technical details of included studies
