# Supplementary material for: Meta‐analysis of peritoneal lavage in appendicectomy
Source: BJS Open. 2018 Nov 29;3(1):24–30. doi: 10.1002/bjs5.50118 (PMC6354188; doi:10.1002/bjs5.50118)
Supplement: Supplementary file 1 — Table S1 Study demographics Table S2 Technical details of included studies [file BJS5-3-24-s001.docx]

**BJS5_50118**

**Meta-analysis of peritoneal lavage in appendicectomy**

**E. Gammeri, T. Petrinic, G. Bond-Smith and A. Gordon-Weeks**

| **Table S1 Study demographics** | | | | | | | | | | | |
| --- | --- | --- | --- | --- | --- | --- | --- | --- | --- | --- | --- |
| **Reference** | **Country** | **Publication date** | **Period** | **Design** | **Total patients** | **Paediatric patients only** | **Male N^o^(%)** | **Laparoscopic N^o^(%)** | **Simple appendicitis N^o^(%)** | **Perforated appendicitis /abscess N^o^(%)** | **Drain used N^o^(%)** |
| **Sun *et al.*^18^** | China | 2017 | 2015–2016 | RCT | 260 | No | 144 (55.4) | 260 (100) | 0 (0) | 260 (100) | 0 (0) |
| **Snow *et al.*^17^** | Australia | 2016 | 2013–2015 | RCT | 81 | No | 52  (64.2) | 81 (100) | 69 (85.2) | 12 (14.8) | 0 (0) |
| **Cho *et al.*^26^** | Korea | 2015 | 2010–2013 | RC | 1817 | No | 925 (50.9) | 1817 (100) | 1321 (72.7) | 496 (27.3) | 592 (32.6) |
| **Hartwich *et al.*^23^** | USA | 2013 | 2005–2011 | RC | 238 | Yes | 126 (52.9) | 123 (51.7) | 0 (0) | 238 (100) | ? |
| **St Peter *et al.*^19^** | USA | 2012 | 2008–2011 | RCT | 220 | Yes | 123 (55.9) | 220 (100) | 0 (0) | 220 (100) | 41 (18.6) |
| **Moore *et al.*^7^** | USA | 2011 | 2007–2008 | RC | 176 | No | ? | 107 (60.8) | 126 (71.6) | 50 (28.4) | ? |
| **Toki *et al.*^24^** | Japan | 1995 | 1984–1993 | RC | 53 | Yes | 22 (41.5) | 0 (0) | 0 (0) | 53 (100) | 24 (45.3) |
| **Stewart and Matheson^25^** | Scotland | 1978 | 1965–1974 | RC | 189 | Yes | ? | 0 (0) | 0 (0) | 189 (100) | 113 (60.8) |

RCT (randomized controlled trial), RC (retrospective cohort)

| **Table S2 Technical details of included studies** | | | | | | | | | | | |
| --- | --- | --- | --- | --- | --- | --- | --- | --- | --- | --- | --- |
| **Reference** | **Laparoscopic** | **Transfixion** | **Appendix removal** | **Irrigation method** | **Standard preop. antibiotics** | **Standard postop. antibiotics** | **Minimum lavage volume (ml)** | **Lavage fluid** | **Method for defining IAA** | **Method for defining WI** | **Length of follow-up (days)** |
| **Sun *et al.*^18^** | Yes | Endoloop | Bag | 4-quadrant | ? | ? | 2000 | Saline | ? | ? | ? |
| **Snow *et al.*^17^** | Yes | Endoloop | ? | Surgeon discretion | Yes | No | 500 | Saline | Clinical, readmission | ? | 43 |
| **Cho *et al.*^26^** | Yes | Lap Loop | Bag | Surgeon discretion | No | Yes | 200 | Saline | ? | ? | 30 |
| **Hartwich *et al.*^23^** | Yes | ? | ? | Surgeon discretion | ? | Yes | ? | Saline | Clinical + CT | Clinical | 7–14 |
| **St Peter *et al.*^19^** | Yes | Staple | Variable | Surgeon discretion | Yes | Yes | 500 | Saline | ? | ? | 14–28 |
| **Moore *et al.*^7^** | Yes | ? | ? | ? | Yes | ? | ? | ? | ? | ? | ? |
| **Toki *et al.*^24^** | No | ? | n.a. | 4-quadrant | 0 | Yes | 1000 | Saline | ? | ? | ? |
| **Stewart and Matheson^25^** | No | ? | n.a. | 4-quadrant | ? | ? | 500 | Noxithioline or tetracycline | ? | ? | ? |

IAA, intra-abdominal abscess; WI, wound infection.
